# Latent profiles of psychological wellbeing and psychological resources in Türkiye: tolerance, patience, compassion satisfaction, psychological flexibility

**DOI:** 10.3389/fpsyg.2025.1723646

**Published:** 2026-01-06

**Authors:** Semra Kiye

**Affiliations:** Department of Guidance and Psychological Counseling, Faculty of Education, Muş Alparslan University, Muş, Türkiye

**Keywords:** latent profile analysis, psychological wellbeing, tolerance, patience, compassion satisfaction, psychological flexibility

## Abstract

**Aims:**

The aim of this study is to identify psychological wellbeing profiles based on the variables of tolerance, patience, compassion satisfaction, and psychological flexibility within the context of Türkiye sample, and to examine whether these profiles differ across demographic subgroups.

**Sample:**

The study was conducted with *N* = 513 voluntary participants living in Türkiye.

**Method:**

Data were collected using the Patience Scale, Compassion Satisfaction Scale, Tolerance Scale, Psychological Flexibility Scale, and Psychological Well-Being Scale. The construct validity of the scales was tested through confirmatory factor analysis (CFA), and the fit indices were found to be at acceptable levels. Internal consistency was confirmed using Cronbach’s alpha coefficients. Latent profile analysis (LPA) was used to identify distinct psychological profiles. The relationship between participant profiles and socio-demographic variables was examined using the chi-square test.

**Results:**

Considering parsimony and theoretical interpretability, a three-cluster solution was selected as the final model. The profiles corresponded to high, moderate, and low levels across all indicators and were labeled as “High,” “Moderate,” and “Low Psychological Well-Being and Psychological Resources.” Correlations among the variables were generally found to be moderately positive. After considering demographic variables, it was found that profiles with higher psychological wellbeing and psychological resources were associated with being female, married, and having a high level of education.

**Conclusion:**

Overall, the findings suggest that psychological wellbeing in Türkiye sample can be better understood through heterogeneous subgroups and provide insights for the design of culturally sensitive interventions and programs.

## 1 Introduction

In recent years, positive psychology and the effects of its concepts on mental health have attracted attention. Psychological wellbeing is one of the most prominent of these concepts, and many studies have been conducted on it. In contrast to subjective wellbeing, psychological wellbeing emphasizes the euodynamic aspect of wellbeing (Keyes et al., 2002). The theoretical framework of psychological wellbeing considers the concept as six basic components and these components are autonomy, environmental mastery, personal growth, positive relationships with others, purpose in life and self-acceptance (Ryff and Keyes, 1995). Psychological wellbeing shows positive relationships with many positive mental health indicators such as resilience (Maor et al., 2022), gratitude (Li et al., 2024), mindfulness, self-compassion (Leung et al., 2024). However, studies on the structure of psychological wellbeing are being carried out in different cultures and samples, both in terms of the aforementioned and other psychological characteristics. In fact, these studies aim to understand the structure of psychological wellbeing in more detail and to evaluate Ryff’s model (Van Dierendonck and Lam, 2023). In this context, the goal of this study is to contribute to the existing literature on psychological wellbeing by examining psychological resources, such as tolerance, patience, compassion satisfaction, and cognitive flexibility, in Türkiye sample.

It is understood that many characteristics related to psychological wellbeing have been examined in studies conducted in Türkiye. Among these, especially cognitive flexibility (Cırcır and Tagay, 2024), stress, depression (Ceri and Cicek, 2021), resilience and life satisfaction (Akbayram and Keten, 2024), and issues related to work and job satisfaction (Tatlıcıoğlu et al., 2024) stand out. In the current study, the relationship between psychological wellbeing and tolerance, patience, compassion satisfaction and psychological flexibility will be examined. Tolerance, patience, and compassion are stated as centuries-old root values in Türkiye culture and are highly valued today (Sezgin and Biçer, 2006; Soyuçok, 2022; Yıldırım and Demirel, 2019). In addition, psychological flexibility, which is considered a relatively new concept in the positive psychology literature, has attracted attention in Türkiye in recent years. When the literature in Türkiye is examined, it is understood that there is a limited number of studies on the relationship between these four traits and psychological wellbeing. For all these reasons, it is important to examine the relationship between these traits and psychological wellbeing in Türkiye. On the other hand, studies have found positive correlations between psychological wellbeing and these traits; tolerance (Bourne et al., 2022; To et al., 2017), patience (Özbay et al., 2024; Turan and Yuce, 2022), compassion satisfaction (Martin-Cuellar et al., 2019) and psychological flexibility (Atasoy Güllü, 2023; Çelik Erdemir, 2024). In this context, it can be argued that supporting the theoretical basis of psychological wellbeing with findings from different samples is important and will contribute to the international literature. Indeed, it is still necessary to support it with different cultures and samples (Van Dierendonck and Lam, 2023). Furthermore, considering these traits alongside psychological wellbeing in the context of individual profiles is thought to significantly contribute to psychological wellbeing literature.

Tolerance is positively related to psychological wellbeing (Bourne et al., 2022; Gündüz and Çağırga, 2023; Yuksel, 2022). A family environment that is tolerant and supportive of the individual’s autonomy contributes to psychological wellbeing (To et al., 2017). Tolerance is refers to respect for differences (Corneo and Jeanne, 2009). Therefore, tolerance involves genuinely positive responses to multiculturalism, diversity and the practices of others (Carew and Shakespeare, 2024). Tolerance can encourage people to live together peacefully and contribute to self-realization. On the other hand, intolerance can prevent the emergence of different talents and tendencies and can be discouraging. An individual is tolerant only to the extent that he or she can respect different characteristics in others in addition to his or her own, and an intolerant person can be indifferent and disrespectful toward other characteristics and lifestyles (Corneo and Jeanne, 2009). It is important to understand the framework of tolerance. For example, tolerance can be shown toward groups linked by religion, belief or ideology. In this case, tolerance refers to the practices of these groups or what they stand for. However, people may also tolerate different practices of friends or family members. Tolerance is an indispensable condition for living with difference (Verkuyten et al., 2022). Research has shown that increased tolerance is associated with an increase in the benevolence of the impersonal world and the general meaningfulness of the world (Galina, 2013). Tolerance plays a moderating role in the relationship between mindfulness and subjective wellbeing (Xu et al., 2016). Also there is a positive relationship between distress tolerance and spiritual wellbeing (Motahari and Torkan, 2025). In addition, tolerance is positively related to patience (Turan and Yuce, 2022). Patience, along with tolerance, is positively related to psychological wellbeing (Özbay et al., 2024).

Patience, as an important determinant of subjective wellbeing (Pfann and Pfann, 2024; Le Pargneux and Zeitoun, 2022), supports individual wellbeing and predicts both hedonic and eudaimonic wellbeing (Schnitker, 2012). Although it is not included in the classification of character strengths and virtues (Peterson and Seligman, 2004), alternative approaches consider patience as a virtue based on empirical results (Lavelock, 2016; Schnitker, 2012). A virtue with more than a thousand years of history, patience is found in the sacred texts of Islam, Christianity and Buddhism (Sweeny, 2024). Today, as in the past, the importance of patience as a virtue or personal trait that supports human development and wellbeing continues to be emphasized (Pianalto, 2016; Schnitker and Emmons, 2007). In its most basic sense, patience is defined as a person’s tendency to wait calmly and serenely in the face of frustration, distress, or pain (Schnitker, 2012). In other words, it is an individual’s willingness to delay gratification (Pfann and Pfann, 2024). In life, individuals often make decisions based on present and future benefits. In most natural situations, waiting for the greater option to come later provides the best long-term outcome. In such situations, the ability to wait for later but greater reward is called patience (Stevens and Stephens, 2008). Patience can be assessed according to life domains, such as patience with life challenges, interpersonal patience, and patience with daily problems (Schnitker, 2012), or as a construct consisting of different components, such as transcendence, forbearance, acceptance, persistence, and delay (Khormaei et al., 2015). Patience can occur in different circumstances and time frames. For example, it may or may not be exhibited in a daily routine such as waiting in traffic, as well as in longer and more challenging situations such as parenting or coping with a serious illness. Although it usually involves a time or waiting component, it can also occur in situations that are not time oriented, such as dealing with a difficult person (Schnitker, 2012). In addition, one of the most important academic and social skills is to take time and strategically exercise patience. In this case, it is said that slowing down is a productive process. In this regard, patience itself is a skill to be learned as a productive learning tool (Roberts, 2013). Patience is positively associated with life satisfaction (Schnitker, 2012; Le Pargneux and Zeitoun, 2022) and wellbeing (Ratchford and Schnitker, 2024). Additionally, negatively associated with depression (Schnitker, 2012), loneliness, stress, anksiety (Ratchford and Schnitker, 2024). Furthermore, positive relationships have been found between patience and positive affect and negative relationships between patience and negative affect (Le Pargneux and Zeitoun, 2022).

Compassion is another psychological trait that is positively associated with positive affect and health indicators and negatively associated with mental distress (Peixoto and Cunha, 2024). One of these positive health indicators is psychological wellbeing. There is a positive correlation between compassion and psychological wellbeing (Sak et al., 2024). Compassion is defined as “an orientation of mind that recognizes suffering and the universality of suffering in human experience and has the capacity to respond to it with kindness, empathy, equanimity and patience” (Feldman and Kuyken, 2011). One study found that compassion has three components. Although these components were identified in different structures, they were classified as noticing, feeling, and responding. Noticing is defined as being aware of an individual’s pain and accepting it cognitively. Feeling is defined as responding emotionally. Responding is defined as being willing to take action to reduce the pain (Kanov et al., 2004). In another definition that defines compassion as cognitive, emotional and behavioral consisting of five components. These components are; “recognizing suffering, understanding the universality of suffering in human experience, feeling empathy for the person suffering and connecting with the distress, tolerating uncomfortable feelings aroused in response to the suffering person so remaining open to and accepting of the person suffering and motivation to act/acting to alleviate suffering.” (Strauss et al., 2016). Compassion satisfaction is defined as a state of sincere satisfaction that an individual feels as a result of compassionate behavior (Stamm, 2002). There are high positive relationships between compassion satisfaction and psychological wellbeing (Martin-Cuellar et al., 2019). Also there are positive relationships between compassion satisfaction and mindfulness (Li et al., 2025), health and wellbeing and demands at work domain (Nadarajan et al., 2025), and resilience (Liu et al., 2025). Additionally, psychological flexibility is another mental health indicator that is positively associated with compassion satisfaction (Garner and Golijani-Moghaddam, 2021).

Psychological flexibility is also an important determinant of psychological wellbeing (Imani et al., 2017; Karadeniz-Özgeniş, 2024). Psychological flexibility significantly explains psychological wellbeing, is effective in identifying individuals at risk of low psychological wellbeing (Guerrini Usubini et al., 2021), and shows an empirically distinct structure from distress (Landi et al., 2021). Psychological flexibility, as one of the main components of mental health, is closely related to resilience and includes many interpersonal and intrapersonal skills (Guerrini Usubini et al., 2021; Kashdan and Rottenberg, 2010). On the other hand, high psychological flexibility is associated with high psychological wellbeing (Atasoy Güllü, 2023; Çelik Erdemir, 2024; Maor et al., 2022) and psychological flexibility has a mediating effect on the effect of positive childhood experiences on subjective wellbeing (Yıldırım, 2023). Individuals with high levels of mindfulness and psychological flexibility tend to have higher psychological wellbeing (Öcel, 2017). Moreover, psychological rigidity negatively predicts psychological wellbeing and as psychological flexibility decreases, psychological wellbeing also decreases (Dalkılınç, 2024). In addition, studies show that psychological flexibility is a moderator of negative effects on learned helplessness and depressive symptoms (Trindade et al., 2020). According to the model of psychological flexibility that forms the basis of acceptance and commitment therapy (ACT), six basic interrelated components are used to increase psychological flexibility. These components are acceptance, defusion, contact with the present moment, self as context, values, and committed action (Hayes, 2004; Hayes et al., 2006). In contrast to psychological flexibility, the main characteristics of psychological rigidity include an inability to adapt to challenging situations or contexts (Waldeck et al., 2021). Similar to psychological flexibility, psychological rigidity consists of six components. These components are weak self-knowledge, experiential avoidance, cognitive fusion, attachment to the conceptualized self, avoidant persistence, and lack of value clarity (Hayes et al., 2006). Psychological flexibility and rigidity are independently related to both hedonic and eudaimonic wellbeing. Accordingly, psychological flexibility and rigidity affect both subjective and psychological wellbeing, and it is recommended to increase the number of studies that address psychological flexibility together with psychological flexibility in order to learn more about the nature of psychological wellbeing (Howell and Demuynck, 2021).

It is considered important to continue research on psychological wellbeing, which is one of the main indicators of mental health. This will contribute to a more detailed understanding of the nature of psychological wellbeing, in addition to evaluations of the psychological wellbeing model. In this context, studies in different cultures and samples are recommended and these studies are expected to strengthen the literature by evaluating Ryff’s model (Van Dierendonck and Lam, 2023). Indeed, this study aims to identify the latent profiles of individuals within Türkiye sample based on the variables of tolerance, patience, compassion satisfaction, and psychological flexibility, which may be associated with psychological wellbeing. In doing so, it seeks to contribute to the literature from different culture and sample. Accordingly, the main objective of the research is to reveal distinct psychological wellbeing profiles that emerge from the combination of tolerance, patience, compassion satisfaction, and psychological flexibility, and to examine whether these profiles differ significantly across subgroups of the sample in terms of gender, marital status, employment status, and educational level.

Research question 1: What psychological wellbeing profiles emerge in Türkiye sample based on the variables of tolerance, patience, compassion satisfaction, and psychological flexibility?

Research question 2: Do the identified psychological wellbeing profiles show statistically significant differences among the subgroups of the sample (gender, marital status, employment status, educational level)?

## 2 Materials and methods

### 2.1 Participants

In this study, maximum variation sampling, a type of purposive sampling method, was employed. This approach was chosen to ensure diversity among participants in terms of gender, age, marital status, educational background, and employment status, with the aim of examining psychological wellbeing across various socio-demographic contexts (Gliner et al., 2011). A total of 513 voluntary participants, all residing in Türkiye and raised within Turkish culture, took part in the study. Participants were informed about the purpose of the research and voluntarily completed the online survey. Participants were informed about the purpose of the research and voluntarily completed the online survey. The data were collected online via Google Forms. This method was primarily chosen to prevent paper and pencil waste and to facilitate the participation process. Participants voluntarily took part in the study through social networks and digital channels. Recruitment was conducted through social networks and digital communication channels, without relying on any institutional affiliation or fixed sampling frame. This strategy enabled the collection of a demographically diverse sample. Of the participants, 71.7% were female and 28.3% were male. The age range extended from 18 to over 40 years, and further demographic breakdowns are provided in Table 1.

**TABLE 1:** Demographic distribution of the sample.

Variable	Level	Frequency	Percentage
Gender	Female	368	71.7
	Male	145	28.3
	Total	513	100
Marital status	Married	175	34.1
	Divorced	13	2.5
	Single	325	63.4
	Total	513	100
Education level	High school or below	42	8.3
	University	394	76.8
	Postgraduate	77	15
	Total	513	100
Employment status	Employed	263	51.3
	Unemployed	250	48.7
	Total	513	100

However, certain imbalances were observed in the sample distribution. For instance, female participants were more willing to participate in the study, whereas male participants appeared more hesitant. This pattern may reflect broader sociocultural dynamics influencing voluntary participation in psychological research. Similarly, the educational level of the sample skewed toward individuals with higher education, particularly university graduates. This tendency may be related to the nature of the study, which was conducted online and addressed psychological concepts that are generally more accessible or appealing to individuals with greater academic exposure. Therefore, while the sample reflects a range of demographic characteristics, the overrepresentation of certain subgroups may limit the generalizability of the findings. These aspects are acknowledged and discussed in the limitations section.

## 3 Measures

### 3.1 Tolerance Scale

The Tolerance Scale was developed by Demirci and Ekşi (2018) with a sample from Türkiye. The scale comprises 6 items and has a unidimensional structure. Items are rated on a five-point Likert scale ranging from “not suitable for me at all (1)” to “completely suitable for me (5).” There are no reverse-coded items in the scale. The minimum score is 6 and the maximum is 30. Higher scores on the Tolerance Scale indicate a higher tendency toward tolerant attitudes and behaviors, such as accepting different perspectives, being patient with others, and showing understanding in interpersonal interactions. Conversely, lower scores suggest lower levels of tolerance and greater difficulty in accepting differences. The scale measures tolerance as a single construct and does not include subdimensions. In this study, confirmatory factor analysis supported the unidimensional structure (χ^2^/df = 3.01, RMSEA = 0.06, GFI = 0.98, CFI = 0.98, NFI = 0.98, IFI = 0.98, SRMR = 0.03). The Cronbach’s alpha coefficient was 0.84, indicating high internal consistency.

### 3.2 Compassion Satisfaction Scale

The Compassion Satisfaction Scale was developed by Nas and Sak (2021). The scale consists of 12 items and has a unidimensional structure. Items are rated on a five-point Likert scale, ranging from “never (1)” to “always (5).” There are no reverse-scored items. The total score ranges from 12 to 60. Higher scores on the scale indicate a greater sense of satisfaction and fulfillment that individuals experience from helping others, expressing kindness, and engaging in compassionate behavior. These scores reflect a person’s internal positive response to performing supportive and caring acts. Lower scores suggest a lower level of satisfaction derived from such actions. Confirmatory factor analysis conducted in this study indicated that the unidimensional model provided a good fit to the data: χ^2^/df = 4.44, RMSEA = 0.08, GFI = 0.93, CFI = 0.97, NFI = 0.96, IFI = 0.97, SRMR = 0.02. In this study, the Cronbach’s alpha coefficient for the scale was found to be 0.70.

### 3.3 Patience Scale

The Patience Scale was developed by Schnitker (2012), and its adaptation to Turkish culture was conducted by Eliüşük and Arslan (2016). The scale consists of 11 items measuring patience across three dimensions: “patience in daily life” (3 items), “patience in the face of life hardships” (3 items), and “interpersonal patience” (5 items). Responses are given on a 7-point Likert scale ranging from “not at all like me (1)” to “exactly like me (7).” All items are positively worded. Higher scores on each subscale and the total scale indicate greater patience, including tolerance for waiting, coping with difficulties calmly, and maintaining composure in interpersonal challenges. Lower scores suggest a lower ability to manage frustration or wait without distress. Confirmatory factor analysis conducted in this study showed that the three-dimensional model provided a good fit to the data: χ^2^/df = 3.52, RMSEA = 0.07, GFI = 0.94, CFI = 0.96, NFI = 0.94, IFI = 0.96, SRMR = 0.04. In this study, the Cronbach’s alpha coefficient for the scale was found to be 0.85.

### 3.4 Psychological Flexibility Scale

The Psychological Flexibility Scale was developed by Francis et al. (2016), and its adaptation to Turkish culture was carried out by Karakuş and Akbay (2020). The scale aims to measure an individual’s ability to act in line with personal values even when facing difficult or distressing experiences. It consists of 28 items rated on a seven-point Likert scale ranging from “strongly disagree (1)” to “strongly agree (7).” The scale is structured around five theoretical subdimensions of psychological flexibility. These include the ability to identify and act in accordance with personal values (values and value-driven action), the capacity to focus attention on the present moment (being present), the willingness to accept thoughts and emotions without trying to change them (acceptance), the perception of the self in a flexible and context-sensitive way (self-as-context), and the ability to relate to thoughts in a more detached and flexible manner (defusion). Several items are reverse-scored: items 2, 3, 5, 6, 8, 14, 18, 20, 22, 23, 24, and 25. Higher scores on the scale indicate greater psychological flexibility, reflecting the individual’s capacity to accept inner experiences, stay present, and behave according to values. Lower scores reflect greater inflexibility and a tendency to avoid or suppress internal experiences in response to distress. The possible total score ranges from 28 to 196. Confirmatory factor analysis conducted in this study indicated that the five-dimensional model provided a good fit to the data: χ^2^/df = 2.53, RMSEA = 0.06, GFI = 0.90, CFI = 0.92, NFI = 0.90, IFI = 0.92, SRMR = 0.06. In this study, the Cronbach’s alpha coefficient for the scale was found to be 0.87.

### 3.5 Psychological Well-Being Scale

The Psychological Well-Being Scale (P-WBS) was developed by Diener et al. (2009), and its adaptation to Turkish culture was conducted by Telef (2013). The scale consists of eight positively worded items and is designed to assess an individual’s psychological wellbeing. It uses a seven-point Likert scale ranging from “strongly disagree (1)” to “strongly agree (7).” Total scores range from 8 to 56. Higher scores on the scale indicate that the individual possesses a high level of psychological resources such as self-respect, optimism, a sense of purpose and meaning in life, and the ability to form rewarding relationships and contribute to others’ happiness. Lower scores may reflect lower levels of these psychological strengths and a reduced sense of wellbeing. The scale has a unidimensional structure and offers a general assessment of psychological wellbeing across several domains. Confirmatory factor analysis conducted in this study indicated that the unidimensional model provided a good fit to the data: χ^2^/df = 3.27, RMSEA = 0.06, GFI = 0.98, CFI = 0.98, NFI = 0.97, IFI = 0.98, SRMR = 0.03. In this study, the Cronbach’s alpha coefficient for the scale was found to be 0.86.

## 4 Data analysis

The aim of this study is to identify psychological wellbeing profiles based on the variables of tolerance, compassion satisfaction, psychological flexibility, and patience within the context of Turkish culture, and to examine whether these profiles differ significantly across demographic subgroups in terms of gender, marital status, employment status, educational level, and age. Data for the study were collected using the Patience Scale, Compassion Satisfaction Scale, Tolerance Scale, Psychological Flexibility Scale, and Psychological Well-Being Scale. These scales are well-established measurement tools that have been used in various studies within the Turkish cultural context. Nevertheless, to verify the validity of these scales in the current study sample, confirmatory factor analysis (CFA) was conducted. Additionally, the reliability of the data obtained through the scales was evaluated by calculating the Cronbach’s alpha coefficient. In the literature, CFA results are typically interpreted based on indices such as χ^2^/df, RMSEA, GFI, CFI, NFI, IFI, and SRMR. According to these indices, χ^2^/df values below 5 (Wheaton et al., 1977), RMSEA values below 0.08 [48], and GFI, CFI, NFI, IFI, and AGFI values of 0.90 or above (Hooper et al., 2008; Bentler, 1995) are considered indicative of good model-data fit. Similarly, SRMR values below 0.08 (Hu and Bentler, 1999) are also regarded as acceptable. In this study, the model fit of the scales was evaluated based on these indices. The reliability coefficient for the scale data was calculated using Cronbach’s alpha. A Cronbach’s alpha coefficient of 0.70 or above is considered sufficient for internal consistency (Bland and Altman, 1997).

After completing the validity and reliability analyses of the scales used in the study, a latent profile analysis (LPA) was conducted in line with the main aim of the research. The LPA aimed to identify latent (hidden) subgroups of individuals who exhibit similar characteristics based on their levels of tolerance, compassion satisfaction, psychological flexibility, and patience, and to define these groups in terms of their psychological wellbeing levels. The analysis was carried out using the Latent Gold 6.0 software.

To evaluate model fit, the following criteria were considered: Bayesian information criterion (BIC), Akaike information criterion (AIC), entropy, classification error (Class. Err), and the number of parameters (Npar). The values obtained for each model are presented in Table 2. Decreases in BIC and AIC values indicate better model fit, while an entropy value close to or above 0.80 supports the reliability of the classification. In addition, the balance of class sizes (percentage distributions) was also taken into account when selecting the optimal model. Based on these criteria, the most appropriate latent profile structure was determined for the study.

**TABLE 2:** Comparative model fit indices for latent profile analysis (LPA).

Model	BIC	AIC	Entropy	Classification error	Number of parameters	Class size
1-Cluster	7,336, 5,526	7,294, 1,498	1.0000	0.0000	10	100%
2-Cluster	6,568, 4,784	6,479, 4,326	0.8237	0.0483	21	55%, 45%
3-Cluster	6,201, 5,751	6,065, 8,863	0.7887	0.0813	32	52%, 35%, 13%
4-Cluster	6,047, 5,391	5,865, 2,073	0.8006	0.0910	43	40%, 33%, 22%, 5%
5-Cluster	6,023, 0259	5,794, 0510	0.7789	0.1258	54	32%, 29%, 21%, 12%, 6%
6-Cluster	5,990, 2,623	5,714, 6,444	0.7908	0.1310	65	28%, 28%, 20%, 13%, 6%, 6%

To examine whether the profiles identified through LPA differed according to demographic variables, chi-square independence tests (χ^2^) were conducted. This test was applied to both binary categorical variables (gender, employment status) and variables with more than two categories (marital status and educational level). When cell frequencies were low, categories were merged or alternative methods (e.g., Fisher’s Exact Test) were employed to ensure statistical validity.

The validity and reliability analyses were performed using SPSS 20.0 and AMOS 24.0, while the latent profile analysis was conducted with Latent Gold 6.0.

## 5 Findings

The mean and standard deviation values of the variables of tolerance, compassion satisfaction, psychological flexibility, patience, and psychological wellbeing, as well as the correlation coefficients showing the relationships between these variables, are presented in Table 3. The strongest relationship was observed between tolerance and patience (*r* = 0.689), while the weakest relationship was found between compassion satisfaction and psychological flexibility (*r* = 0.269). The results indicate that there is generally a moderate positive relationship between the variables, and the variables exhibit consistent relationships with one another.

**TABLE 3:** Descriptive statistics and correlation coefficients for the variables.

Variable	Patience	Tolerance	Compassion satisfaction	Psychological flexibility	Mean	SD	Min	Max
Patience	–				40.49	7.94	11	77
Tolerance	0.689***	–			24.10	4.37	6	30
Compassion satisfaction	0.440***	0.502***	–		53.86	8.37	12	60
Psychological flexibility	0.475***	0.342***	0.269***	–	128.15	17.21	28	196
Psychological wellbeing	0.429***	0.413***	0.454***	0.507***	41.85	8.15	8	56

*N* = 513. All correlations are Pearson coefficients (1-tailed).

***p < 0.001. Minimum and maximum values are based on theoretical scale ranges.

After examining the relationships among the variables, latent profile analysis (LPA) was conducted on five psychosocial variables — psychological wellbeing, patience, psychological flexibility, compassion satisfaction, and tolerance. Models with one to six latent classes were compared using LPA. As shown in Table 2, the BIC and AIC values decreased as the number of classes increased. However, model interpretability and balance in class sizes were also taken into consideration. Although the four-class solution (BIC = 6,047.54; AIC = 5,865.21; Entropy = 0.80) appeared statistically adequate, the final class contained only 5% of participants, which reduced the stability of the model. In the five- and six-class models, class size imbalance increased, and classification error also rose. In contrast, the three-class model (BIC = 6,201.58; AIC = 6,065.88; Entropy = 0.79; Classification Error = 0.08) provided a statistically satisfactory fit and yielded three meaningful and interpretable profiles, representing 52, 35, and 13% of the sample, respectively. Therefore, considering parsimony and theoretical interpretability, the three-class solution was selected as the final model.

**FIGURE 1:**
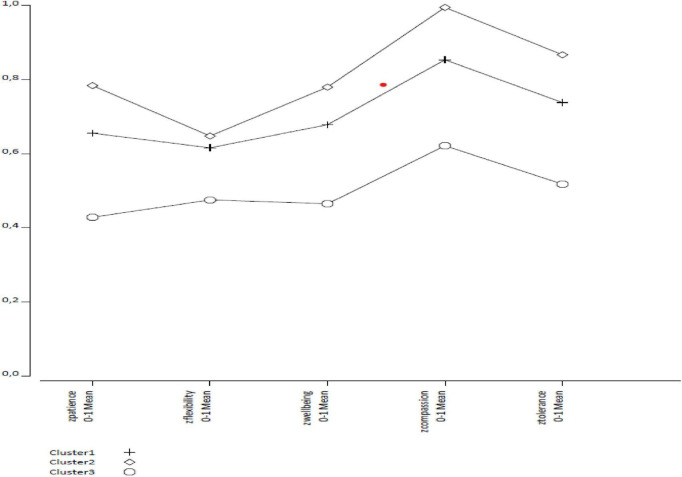
Profile-based mean values of psychological wellbeing and psychological resources variables according to latent profile analysis.

As a result of the analysis, the three-class solution was examined in more detail, and the profiles were labeled accordingly (see Figure 1). The first profile, comprising more than half of the sample, consisted of participants who exhibited moderate scores across all variables; this group was labeled “Moderate Psychological Well-Being and Psychological Resources.” The second profile included participants who displayed high scores on all dimensions, with particularly high levels of compassion satisfaction and psychological wellbeing; this group was defined as “High Psychological Well-Being and Psychological Resources.” The third profile consisted of participants who showed low scores on all indicators and could be considered a psychologically at-risk group; this group was labeled “Low Psychological Well-Being and Psychological Resources.” This three-profile structure demonstrates that participants can be meaningfully distinguished based on their psychosocial characteristics, and that latent profile analysis (LPA) effectively identifies heterogeneous subgroups in terms of psychological wellbeing levels.

Finally, the study examined whether the psychological wellbeing profiles identified through latent profile analysis (LPA) differed significantly across the demographic subgroups of the sample (gender, marital status, employment status, and educational level). For this purpose, chi-square tests were conducted for variables with two categories (gender, employment status) to evaluate whether the distributions of the profiles differed statistically across these variables. For variables with more than two categories (marital status and educational level), chi-square test results were again examined, and class frequencies were compared to test for significant differences. The results of the analysis revealed that psychological wellbeing profiles differed significantly according to certain demographic variables. The findings are presented in Table 4.

**TABLE 4:** Chi-square test results regarding the relationship between individual characteristics and latent profiles.

Variable	Level	Moderate psychological wellbeing and psychological resources (%) 1	High psychological wellbeing and psychological resources (%) 2	Low psychological wellbeing and psychological resources (%) 3	χ^2^	SD	*P*
Gender	Male	46.9%	31.7%	21.4%	6.04	2	0.049
	Female	50.8%	36.4%	12.8%			
Marital status	Single	50.2%	30.8%	19.1%	15.82	4	0.003
	Divorced	38.5%	38.5%	23.1%			
	Married	49.7%	35.1%	15.2%			
Educational level	High School	21.4%	45.2%	33.3%	29.49	4	< 0.001
	University	50.5%	33.5%	16.0%			
	Postgraduate	61.0%	37.7%	1.3%			
Employment status	Employed	52.5%	34.6%	12.9%	2.71	2	0.258
	Unemployed	49.7%	35.1%	15.2%			

According to the chi-square independence test results, levels of psychological wellbeing and psychological resources differed significantly across gender, marital status, and educational level. From the perspective of gender, the proportion of women in the moderate psychological wellbeing profile (50.8%) was higher than that of men (46.9%). In contrast, men were more likely to fall into the low-level group (21.4%) (χ^2^ = 6.04, *p* = 0.049). Regarding marital status, married individuals showed a more balanced distribution across the high (35.1%) and moderate (49.7%) levels; this indicates that, compared to single individuals (30.8% and 50.2%, respectively), married participants generally exhibited higher levels of psychological wellbeing (χ^2^ = 15.82, *p* = 0.003). In terms of educational level, participants with postgraduate (61.0%) and university (50.5%) degrees were much more likely to belong to the moderate psychological wellbeing profile compared to high school graduates (21.4%) (χ^2^ = 29.49, *p* < 0.001). In contrast, no significant difference was observed between groups in terms of employment status (χ^2^ = 2.71, *p* = 0.258). Overall, these findings suggest that women, married individuals, and those with higher educational attainment tend to be more advantaged in terms of psychological wellbeing and psychological resources.

## 6 Conclusion and discussion

In this study, different psychological wellbeing profiles emerging from the combination of tolerance, patience, compassion satisfaction, and psychological flexibility in Türkiye sample were examined. We also examined whether the identified psychological wellbeing profiles differed among subgroups based on gender, marital status, employment status, and education level. The results indicate that psychological wellbeing is positively related to all other traits. Furthermore in the context of the first research question of the study, three profiles were identified: (1) moderate psychological wellbeing and psychological resources (52%), (2) high psychological wellbeing and psychological resources (35%), (3) low psychological wellbeing and psychological resources (13%). Furthermore, in regard to the second research question, levels of psychological wellbeing and psychological resources differ according to gender, marital status, and level of education. Women exhibit significantly higher levels of moderate and high psychological wellbeing and psychological resources than men. Married individuals also exhibit higher levels of psychological wellbeing and resources than single individuals. Additionally, high school graduates have lower levels of psychological wellbeing and resources than undergraduate and graduate students. There is no significant difference by employment status.

The current study found that all these psychological traits were positively correlated with each other. This finding is supported by the literature (Dalkılınç, 2024; Maor et al., 2022; Le Pargneux and Zeitoun, 2022; Martin-Cuellar et al., 2019; Özbay et al., 2024). However, most participants fall into the first profile, which indicates moderate levels of psychological well-being and psychological resources. Similar findings have emerged from studies conducted with different samples, indicating that the majority of participants exhibit moderate levels of positive mental health indicators (Ceri and Cicek, 2021; Kiye et al., 2024; Liu et al., 2025; Nadarajan et al., 2025). In addition, this study shows that people with moderate levels of psychological wellbeing and psychological resources are mostly women and highly educated individuals. While some studies in the literature are consistent with this finding regarding psychological wellbeing and gender (Gündüz and Çağırga, 2023; Sak et al., 2024; Yuksel, 2022), others show no difference (Ceri and Cicek, 2021; Polat, 2024). This result in this sample may be related to the fact that psychological wellbeing can be explained by many different conditions and characteristics. On the other hand, the fact that people with higher levels of education mostly fit this profile may be related to the idea that, as one’s level of education increases, one has a more exploratory attitude toward oneself and realizes one’s internal and external resources in greater detail. Additionally, there are also studies showing that education level does not make a difference for psychological wellbeing and other psychological traits (Kiye et al., 2024; Sak et al., 2024). These different results may highlight the need for further research into the nature of psychological wellbeing.

A significant proportion of participants in the second profile exhibit high psychological well-being and psychological resources. Although this group is smaller than the first group, it still includes more than one-third of the participants. Research supports this finding, showing that high psychological wellbeing is associated with high psychological resources (Atasoy Güllü, 2023; Çelik Erdemir, 2024; Maor et al., 2022; Mendes et al., 2023). It was also found that individuals with this profile were female, highly educated, and married. These results are consistent with previous studies (Gündüz and Çağırga, 2023; Sak et al., 2024). Family life satisfaction is an important indicator of psychological wellbeing (Kiye and Demir, 2025) the higher satisfaction of married individuals may increase their overall wellbeing. In addition to these results, studies have shown that being female (Ceri and Cicek, 2021) or highly educated and married (Kiye et al., 2024) does not significantly impact psychological wellbeing. This may indicate the need for further, more detailed research on the psychological wellbeing model, as previously mentioned.

The third profile, low psychological wellbeing and psychological resources, comprises a relatively small proportion of participants compared to the other groups. However, due to low psychological wellbeing and psychological resources, individuals in this profile may be considered a risk group. In fact, it is understood from the literature that those with low levels of psychological wellbeing and psychological resources are individuals in the risk group, such as immigrants (Coşkun and Kiye, 2025). Research shows that psychological wellbeing and other psychological resources are negatively associated with depression (Engüzel, 2024; Schnitker, 2012), doomscrolling (Kiye et al., 2024), as well as with loneliness, stress, and anxiety (Ratchford and Schnitker, 2024), psychological distress (Peixoto and Cunha, 2024). Additionally, positive correlations have been found between these psychological traits and positive affect, while negative correlations have been found with negative affect (Le Pargneux and Zeitoun, 2022; Peixoto and Cunha, 2024). These results indicate that preventive interventions are necessary for individuals with this profile. It was also found that most of those in this profile were male, had a low level of education, and were single. Based on these results, it is crucial to develop intervention programs for unmarried men with low levels of education. Including modules that incorporate characteristics such as tolerance, patience, compassion, satisfaction, and psychological flexibility can increase the effectiveness of interventions designed to improve the psychological wellbeing of this group.

In conclusion these findings, observed in a different sample, significantly contribute to the literature on psychological wellbeing. In the Türkiye sample, individuals with high wellbeing were found to have higher levels of tolerance, patience, compassion, contentment, and flexibility. While most participants had moderate to high levels of psychological wellbeing and other psychological resources, a smaller minority had lower levels of these resources. Additionally, it was determined that 13% of the participants were individuals with low psychological wellbeing and resources. Most of these participants were single men with low education levels. The importance of developing intervention programs to address this issue was emphasized.

### 6.1 Limitations and recommendations

This study examines the relationship between psychological wellbeing and four psychological traits: psychological flexibility, compassion satisfaction, tolerance, and patience. In accordance with the principle of scientific transparency, the study’s limitations are outlined below.

First, the study employed a cross-sectional and correlational design, which does not allow for causal inferences between variables. To obtain more detailed results, further research should be conducted in the form of longitudinal studies.

Second, the study relied exclusively on self-reported instruments, which may introduce various sources of bias such as social desirability or common method variance. Although these instruments have previously demonstrated acceptable psychometric properties in Türkiye samples, the use of a single data source and format raises the possibility of inflated correlations due to method variance. Future studies could benefit from incorporating procedural remedies such as temporal separation of measurement, use of different response formats, or multi-source data collection, as recommended by Podsakoff et al. (2003). Additionally, given the subjective and introspective nature of the psychological traits assessed in this study (e.g., psychological flexibility, compassion satisfaction, tolerance), self-report instruments are widely considered valid and appropriate tools in behavioral and psychological research. Moreover, previous research has demonstrated that self-ratings can reflect valid internal states, despite occasional bias (MacIntyre et al., 1997).

Third, the study utilized a maximum variation sampling method to ensure demographic diversity in terms of gender, age, marital status, education level, and employment status. Additionally, since this research was conducted on social media, the sample may be biased because not all segments of society use social media equally. However, since participation was voluntary, some subgroups were more inclined to participate than others. The participants in this study were predominantly female and highly educated. This is an important detail that should not be overlooked. As a result, certain demographic groups may be overrepresented or underrepresented in the sample, which requires careful interpretation regarding the generalizability of the findings.

Fourth high correlations between some variables (e.g., patience and tolerance) were observed, which is common in social science research due to the overlapping nature of psychological constructs. This should be considered when interpreting the findings.

These limitations are not intended to undermine the validity of the study, but rather to provide context and guide future research. Within the scope of its sample and model, the current study offers strong and meaningful contributions to understanding psychological wellbeing in a culturally relevant framework.

## Data Availability

The original contributions presented in this study are included in this article/supplementary material, further inquiries can be directed to the corresponding author.
